# Evidence-based care in high- and low-risk groups following whiplash injury: a multi-centre inception cohort study

**DOI:** 10.1186/s12913-019-4623-y

**Published:** 2019-11-06

**Authors:** Alexandra Griffin, Jagnoor Jagnoor, Mohit Arora, Ian D. Cameron, Annette Kifley, Michele Sterling, Justin Kenardy, Trudy Rebbeck

**Affiliations:** 1 Faculty of Health Sciences, The University of Sydney, 75 East St, Lidcombe, NSW Australia; 2 John Walsh Centre for Rehabilitation Research, The University of Sydney, Kolling Institute, St Leonards, NSW Australia; 3 NHMRC Centre of Research Excellence in Road Traffic Injury, The University of Queensland, Brisbane, QLD Australia; 4 Recover Injury Research Centre, The University of Queensland, Level 7, UQ Oral Health Centre, Herston, QLD Australia; 5 The George Institute for Global Health, The University of Sydney, Level 5, 1 King St, Newtown, NSW Australia

**Keywords:** Whiplash injuries, Evidence-based health care, Clinical practice guidelines, Practice guidelines, Cohort

## Abstract

**Background:**

Studies aimed at improving the provision of evidence-based care (EBC) for the management of acute whiplash injuries have been largely successful. However, whether EBC is broadly provided and whether delivery of EBC varies based on risk of non-recovery, is uncertain. Receiving EBC should improve recovery, though this relationship has yet to be established. Further, mitigating the effect of EBC is the relationship with the practitioner, a phenomenon poorly understood in WAD. This study aimed to determine the proportion of individuals with whiplash, at differing baseline risk levels, receiving EBC. This study also aimed to determine whether receiving EBC and the therapeutic relationship were associated with recovery at 3 months post injury.

**Methods:**

Participants with acute whiplash were recruited from public hospital emergency departments, private physiotherapy practices, and State Insurance Regulatory Authority (SIRA) databases. Participants completed questionnaires at baseline (demographics, risk of non-recovery) and 3-months (treatment received, risk identification, therapeutic relationship) post injury. Primary health care providers (HCPs) treating these participants also completed questionnaires at 3-months. Recovery was defined as neck disability index ≤4/50 and global perceived effect of ≥4/5.

**Results:**

Two-hundred and twenty-eight people with acute whiplash, and 53 primary care practitioners were recruited. The majority of the cohort reported receiving EBC, with correct application of the Canadian C-spine rule (74%), and provision of active treatments (e.g. 89% receiving advice) high. Non-recommended (passive) treatments were also received by a large proportion of the cohort (e.g. 50% receiving massage). The therapeutic relationship was associated with higher odds of recovery, which was potentially clinically significant (OR 1.34, 95% CI 1.18–1.62). EBC was not significantly associated with recovery.

**Conclusions:**

Guideline-based knowledge and practice has largely been retained from previous implementation strategies. However, recommendations for routine risk identification and tailored management, and reduction in the provision of passive treatment have not. The therapeutic relationship was identified as one of several important predictors of recovery, suggesting that clinicians must develop rapport and understanding with their patients to improve the likelihood of recovery.

## Background

Recovery following whiplash injury, if it is to occur, will occur for the most part within the first 3 months post injury [1]. Current estimates suggest that approximately 50% of individuals will recover by 3 months post injury, whilst the remainder will experience mild to moderate long-term disability [1–3]. Since the majority of recovery in Australian cohorts occurs within the first 3 months following injury [1, 4], guidelines for the management of acute whiplash associated disorders (WAD) have an important role. In New South Wales (NSW), Australia, clinical practice guidelines for the management of acute WAD were developed with the purpose of improving management and patient outcomes. Recommendations were based on the National Health and Medical Research Council (NHMRC) grade of evidence [5]. Key recommendations were identified for implementation from these guidelines and included: (1) Appropriate imaging and selection of active treatments, (2) A reduction in the selection of passive treatments, and (3) An improvement in the process of care [6]. Whilst implementation of these recommendations in previous work [7–9] has improved the provision of appropriate care, little is known as to whether the provision of EBC and the process of care are maintained and associated with improved recovery. The translation of evidence to consumers, particularly patients and clinicians, via evidence-based, specific, and simple recommendations is essential for achieving compliance [10, 11].

The first key recommendation identified from the Australian whiplash guidelines was the appropriate application of the Canadian C-Spine rule. The Canadian C-Spine rule is a widely validated rule used to assist clinicians in determining whether an x-ray is required to confirm the diagnosis of a clinically important cervical injury [12]. The application of the rule involves working though a series of questions regarding risk factors for clinically important cervical spine injuries (i.e. cervical fracture or dislocation). Risk factors are weighted and presented in a flow-chart format. With correct application, the Canadian C-Spine rule is highly sensitive (range 0.90–1.00), and has the potential to reduce unnecessary exposure to radiation by as much as 42% [13]. However at present, the rate of unnecessary imaging in whiplash cohorts is unknown.

The provision of active treatments, such as advice and neck exercises, and the reduction of passive treatments with little or no evidence supporting their use, was also a key recommendation. The relative efficacy of active versus passive treatments is well established, [14–16] and whilst previous studies have demonstrated good uptake of the provision of active treatments, [7–9] to date it is unknown whether the provision of passive treatments has reduced.

The final key recommendation from the Australian whiplash guidelines was a differential process of management based on risk of non-recovery. Individuals at risk of non-recovery should be identified early, using a recommended prognostic tool, to facilitate timely and appropriate multidisciplinary management. Recommended prognostic tools include measures of pain intensity using the visual analogue scale (VAS), or the numeric rating scale (NRS), and measures of neck pain-related disability using the neck disability index (NDI). Scores of > 5/10 on the NRS or VAS, and > 15/50 on the NDI are associated with poorer recovery [6]. Additionally, a clinical prediction rule (CPR) to estimate risk of non-recovery in WAD has been developed [17] and validated [18]. This rule has a positive predictive value (PPV) of 71% in predicting individuals: (a) at risk of moderate to severe disability, and (b) likely to make full recovery, at 12 months post injury. The CPR incorporates age, the NDI and the hyperarousal sub scale of the post-traumatic diagnostic scale (PDS). Since its recent development, it is unknown whether clinicians are utilising this CPR, or any of the other prognostic indicators, to correctly identify individuals at risk of non-recovery, and tailor management accordingly.

Following early identification of risk, the guidelines recommend differential management. For example, it is recommended that individuals at low-risk of non-recovery have minimal care, and individuals at high risk be referred to WAD specialists, including rehabilitation physicians, specialist physiotherapists, psychologists and occupational physicians [6]. The provision of care based on risk has shown effectiveness in other musculoskeletal conditions, including low back pain [19]. It is proposed [20] yet unknown whether a similar risk stratification approach will result in improved health outcomes for whiplash. Examining current practice in regards to differential management strategies for WAD, based on risk, is an important starting point in this process.

Finally the relevance of the provision of EBC may be important only if it relates to recovery. Given recovery following whiplash injury is poor, it is important to establish the relationship between provision of EBC and recovery. Mitigating the effect of EBC is how it is delivered, namely the relationship with the practitioner. The therapeutic relationship refers to the sense of trust, warmth and support between patient and clinician, [21] and is positively associated with improved outcomes in low back pain [22] and mental health [23] populations. Whilst well established in psychotherapy research, the role of the therapeutic relationship in mediating outcomes in WAD is unknown. The therapeutic relationship, together with the provision of EBC, may have a significant influence on outcomes following WAD, though this is currently unknown. Therefore, the aims of this study were to determine the proportion of people with WAD receiving EBC, and whether baseline risk level, receiving EBC and the strength of the therapeutic relationship, were associated with recovery at 3 months post injury.

## Methods

### Study design

This study was a prospective, multi-centre inception cohort study of patients with acute WAD.

### Participants

Participants included individuals with whiplash [*participants with whiplash*], and their primary HCPs. Whiplash is defined, according to the Quebeck Task Force, as an acceleration-deceleration mechanism of energy transfer to the cervical spine, commonly resulting in a variety of clinical manifestations (whiplash-associated disorders) [24]. Participants were eligible if they reported neck pain following a motor vehicle crash, consistent with a whiplash injury of grade I-III, [24] were at least 17 years of age, and were within 28 days of injury. Participants were excluded if they had a pre-existing cognitive impairment affecting ability to consent, or had suffered severe physical injury (e.g. fracture (WAD IV), spinal cord injury) or psychological trauma (e.g. death of a family member). Complete inclusion and exclusion criteria are described elsewhere [25].

Primary HCPs were included if they were a primary care practitioner treating a participant with whiplash, were contactable, and provided informed consent.

### Setting

Participants with whiplash were recruited from public hospital emergency departments, private physiotherapy practices and State Insurance Regulatory Authority (SIRA) databases in NSW, Australia. The SIRA databases include the Personal Injury Register (PIR), where it is mandatory for insurance claims to be reported in NSW, and the Claims Advisory Service (CAS) database, which provides support for the assessment and resolution of motor vehicle accident compensation claims.

Participants with whiplash were contacted by telephone by the research team. Eligibility was established and informed consent obtained. Participants with whiplash completed baseline and 3-month follow-up questionnaires. At 3 months, participants were asked to identify their primary HCP, defined as their main primary health care provider following injury. This study was approved by the Sydney Local Health District Ethics Committee; reference number HREC/13/CRGH/67.

### Baseline questionnaires

Baseline questionnaires assessed demographic information, pain and disability, risk status, psychological factors and general health. Demographic data captured included age, gender, weight, height, martial status, education level and country of birth.

Pain was assessed at baseline using the NRS. The NRS is frequently used in healthcare settings, [26, 27] and ranges from 0/10 (no pain) to 10/10 (worst pain possible). Pain catastrophising was assessed using the pain catastrophising scale (PCS), a 13-item questionnaire with scores ≥25/52 representing a clinically significant level of pain catastrophising [28]. Patient self-reported disability was assessed using the NDI [29]. The NDI is widely used in whiplash cohorts [30–32] and ranges from 0/50 (no disability due to neck pain) to 50/50 (maximum disability due to neck pain).

Baseline risk status was assessed using a validated CPR [18]. This CPR uses baseline NDI, age and the hyperarousal sub-scale of the Post-traumatic Diagnostic scale (PDS) to predict the likelihood of recovery at 12 months post injury. Using the CPR, participants are classified as low-, medium- or high-risk of ongoing pain and disability.

Psychological factors assessed at baseline included post-traumatic stress symptoms, negative expectations of recovery, and depression, anxiety and stress. These constructs were chosen due to their association with non-recovery after whiplash [6]. Post-traumatic stress symptoms were measured using the revised Impact of Events Scale (IES-R), [33, 34] with higher scores indicative of greater distress and predictive of increased risk of non-recovery [6]. Expectations of recovery were assessed using question 7 within the short-form Orebro Musculoskeletal Pain Questionnaire (OMPQ; range 0/10: no perceived risk of pain becoming persistent to 10/10 very large perceived risk of pain becoming persistent) [35]. The Depression, anxiety and stress scale (DASS) [36] was used to evaluate these constructs, with depression associated with poor outcomes after whiplash [6].

General health questionnaires assessed at baseline included the Short Form-12 (SF-12; version 1) Health Survey and the European Quality of Life – 5 Dimensions – 3 levels (EQ5D3L) questionnaire.

### Three-month follow-up questionnaires

Participants with whiplash completed three questionnaires at follow-up, assessing treatment received, recovery, and the therapeutic relationship (Additional file 1). The questionnaire regarding treatment received included questions on whether the Canadian C-spine rule was applied correctly, the treatment received, and if and when referral to specialist clinicians occurred.

Recovery at 3 months was assessed using the NDI, and the Global Perceived Effect (GPE) scale. The GPE quantifies a patient’s *global* improvement or deterioration over time, measured on an 11-point scale ranging from − 5/5 (vastly worse) to + 5/5 (completely recovered) [37]. Therapeutic relationship was assessed using a modified version of the Working Alliance Theory of Change Inventory (WATOCI) scale [38]. Scores ranged from 9/45 (very poor therapeutic relationship) to 45/45 (excellent therapeutic relationship).

Primary HCPs completed similar questionnaires regarding treatment provided and therapeutic relationship. Practitioners were additionally asked to self-nominate the perceived risk status of their patient, as either low-, medium- or high-risk of non-recovery.

*The Whiplash Evidence-based Care tool* (WAD-Evidence tool) was derived for use in the study, and based on previous questionnaires used to measure adherence to Australian whiplash guidelines [8, 9]. It was modified to reflect the key recommendations identified from the 2014 edition [6]. Recommendations related to both *content* and *process* for management. *Content*-based recommendations included the correct application of the Canadian C-Spine Rule and the provision of recommended activating treatments. These treatments include advice and exercises. *Process*-based recommendations referred to risk identification and referral decisions. Scores were assigned for compliance with recommendations, with higher scores assigned if the practice was compliant with Grade A/B recommendations [39, 40] and negative scores if the practice was non-compliant with Grade A/B recommendations. The scoring system is presented in detail in Table 1. Scores were summed to give a total WAD-Evidence tool score (range − 85 to 165), with higher scores reflecting greater compliance with EBC.

**Table 1:** Whiplash Evidence Based Care Tool (“WAD-Evidence tool”). (Range: − 85 (poorest compliance) to + 165 (maximum compliance) with evidence based recommendations)

Key recommendation	Measured by	NHMRC Grade of Recommendation:	Compliant	Non-compliant
PART 1: Content				
Apply the Canadian C-Spine rule to determine whether an x-ray is required to confirm suspected fracture or dislocation	Applied correctly	A	+ 5	−5
	Applied incorrectly	A		
Identify and provide evidence-based treatment	Recommended treatments			
	Advice	B	+ 20	0
	Exercise	B	+ 20	0
	Pharmacology	Consensus	+ 20	0
	Treatments not routinely recommended			
	Manual therapy & manipulation	C	+ 10	0
	Acupuncture	D	+ 10	0
	Surgery	Consensus	+ 10	0
	Treatments with no evidence for or against their use			
	Pilates	Consensus	0	−5
	Massage	Consensus	0	−5
	Cervical pillows	Consensus	0	−5
	Electrotherapy	Consensus	0	−5
	Treatments not routinely recommended			
	Injections	Consensus	0	−20
	Collar	A	0	−20
PART 2: Process				
Provide the appropriate number of treatments	Number of treatments by risk level			
	Low-risk	N/A		
	0–3		+ 20	0
	4–12		0	−5
	13–20		0	−10
	> 20		0	−20
	High-risk	N/A		
	0–5		0	−5
	6–16		+ 20	0
	17–24		0	−5
	25–32		0	−10
	> 32		0	−20
Consider specialist referral if at-risk of non-recovery	Low-risk (no referral) High-risk (referral)	N/A	+ 20	0
	Time to referral < 6 weeks	N/A	+ 10	0
	Referred to WAD specialist	N/A	+ 20	0
	Referred to other specialist	N/A	+ 5	0
Total			165	−85

### Outcomes

The primary outcome at 3-month follow-up was recovery measured by NDI and GPE. Recovery was defined as a score of 4 or more on the 11-point GPE, and a score of ≤4/50 on the NDI, based on consensus decision and previous definitions [17, 37, 41]. We also investigated the proportion of individuals experiencing clinically significant improvements in neck disability (defined as mild to no disability; NDI ≤ 29/50 [41]) and GPE (≥ 2-point increase on 11-point scale [37]).

Additional secondary outcomes included the proportion of participants with whiplash receiving EBC via the WAD-Evidence tool, and the therapeutic relationship, according to primary HCP report. Correct identification of risk level of participants with whiplash and the appropriate decision-making processed around referral on the part of the primary HCP were also measured.

### Sample size

A post hoc sample size calculation showed that the present design would provide 83% power to detect recovery at *α* = 0.05.

### Statistical analysis

Statistical analyses were performed using SPSS v22 [42]. Baseline characteristics and 3-month variables of interest were summarised using descriptive statistics. The distribution of these data were assessed using the Shaprio-Wilk test and between-group comparisons were performed for normally-distributed data using t-tests or ANOVA. The Kruskal-Wallis non-parametric test was used where data were not normally distributed. Chi-Square analyses were used to assess associations between categorical variables of interest. The sample size (n) and standard deviation (SD) were also presented for each variable of interest. Where significant differences were found overall among multiple groups, post-hoc tests were used to identify which groups differed. For between-group comparisons using ANOVA, the LSD post hoc test was used.

A separate logistic regression model was derived for each outcome through a forward-stepwise process to assess the individual contribution of each predictor variable to the model.

Both known (e.g. CPR, expectations of recovery, baseline pain intensity) and potential (e.g. WAD-Evidence tool score, therapeutic relationship) predictors of recovery were assessed.

Predictors significantly (*p* <  0.05) associated with recovery, alone, were ranked in order of significance. Odds ratios with 95% confidence intervals, Nagelkerke R^2^ and *p*-values were reported. These predictors were then combined sequentially in a forward stepwise manner, retaining only those that continued to contribute significantly to the model. A final ‘reduced’ model, containing only those variables with significant contributions, was created for each outcome. The potential predictors of therapeutic relationship and WAD-Evidence tool score were then added to each model to assess their individual contribution to the model.

In order to determine whether the two predictor variables, in combination, had a different contribution to the model than they would in isolation, their interaction was assessed. A model was run in the form of *y* = therapeutic relationship + WAD-Evidence tool score + therapeutic relationship*WAD-Evidence tool score. The significance of the interaction term (therapeutic relationship*WAD-Evidence tool score) in this model was then assessed. Mixed effects models were not suitable for use in the case of these data.

## Results

**Fig. 1:**
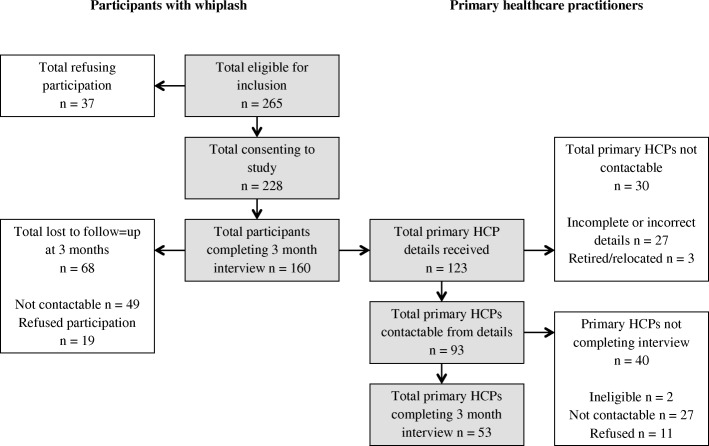
Flow of participants through the study

A total of 228 participants with whiplash, and 53 primary HCPs, were recruited. Three-month data collection was completed for 160 participants, with 68 participants with whiplash lost to follow-up. Contact details were received for 123 primary HCPs, with 93 contactable and 53 consenting to participate (Fig. 1). Baseline characteristics and outcomes are 2 months for participants with whiplash are presented in Tables 2 and 3.

**Table 2:** Baseline characteristics

Variables	N	All participants (*n* = 215)	Lost to follow-up (*n* = 53)	*P*-value	Low risk (*n* = 35)	Medium risk (*n* = 106)	High risk (*n* = 66)	*P*-value
Age, mean (SD), in years	205	42 (16)	41(15)	*0.597*	26 (6)****	41 (17)****	53 (12)****	*< 0.001*
Gender, n (%)	198							
Male		89 (45)	21 (44)	*0.848*	12 (38)	43 (43)	32 (51)	*0.432*
Female		109 (55)	27 (56)		20 (63)	56 (57)	31 (49)	
Body Mass Index, mean (SD)	198	27.5 (7.4)	27.6 (7.1)	*0.898*	25.6 (6.2)	26.9 (6.3)	29.4 (9.1)*	*0.035*
Continent of birth, n (%)	204							
Australia		128 (60)	30 (63)	*0.952*	26 (77)	65 (64)	32 (51)	*0.114*
Europe		19 (9)	5 (10)		1 (3)	11 (11)	7 (11)	
Other		57 (27)	13 (27)		7 (21)	26 (26)	24 (38)	
Marital status, n (%)	204							
Never married		63 (29)	19 (40)	*0.089*	21 (62)	34 (33)	8 (13)	*< 0.001*
Married or de facto		113 (53)	20 (42)		11 (32)	57 (55)	43 (68)*	
Divorced, widowed or separated		28 (13)	9 (19)		2 (6)	12 (12)	12 (19)	
Position in crash, n (%)	204							
Driver		136 (63)	33 (69)	*0.979*	21 (62)	78 (76)	34 (54)	*0.034*
Passenger		32 (15)	7 (15)		7 (21)	11 (11)**	14 (22)	
Motorcyclist		21 (10)	5 (10)		3 (9)	11 (11)	6 (10)	
Bicyclist or pedestrian		15 (7)	3 (6)		3 (9)	3 (3)	9 (14)	
Hospital presentation, mean (SD), days	204	189 (88)	44 (92)	*0.766*	29 (85)	95 (92)	62 (98)*	*0.049*
Hospital LOS, mean (SD), in days	101	3.1 (3.4)	2.04 (1.5)	0.009*	2.07 (1.3)	3.1 (3.6)	3.6 (3.7)	*0.412*
Employment, mean (SD)	204							
Paid work		125 (58)	28 (58)	*0.328*	26 (77)	68 (66)	28 (44)	*0.001*
Self-employed		29 (14)	11 (23)		3 (9)	12 (12)	14 (22)	
Student		11 (5)	2 (4)		4 (12)	7 (7)	0 (0)*	
Retired		18 (8)	4 (8)		0 (0)	7 (7)	11 (18)	
Other		21 (10)	3 (6)		1 (3)	9 (9)	10 (16)	
Occupation type	154							
Professional		52 (24)	13 (33)	*0.829*	13 (45)	22 (28)	16 (38)	*0.292*
Clerical and administrative		21 (10)	4 (10)		3 (10)	14 (18)	4 (10)	
Technical and trades		18 (8)	3 (8)		1 (3)	12 (15)	5 (12)	
Manager		22 (10)	7 (18)		2 (7)	13 (17)	6 (14)	
Community and personal services		19 (9)	5 (13)		7 (24)	7 (9)	5 (12)	
Other		22 (10)	7 (18)		3 (10)	12 (15)	6 (14)	
Pain/ Disability								
NDI total score (0–50/50)	209	21 (9)	21 (10)	*0.875*	11 (4)****	20 (9)****	28 (5)****	*< 0.001*
Pain during past week (NRS: 0–10/10)	210	7 (2)	7 (2)	*0.618*	6 (2)	6 (2)	8 (2)*	*< 0.001*
PCS total score (0–52/52)	209	22 (14)	24 (14)	*0.302*	16 (10)	20 (13)	30 (14)*	*< 0.001*
PCS- Rumination (0–16/16)	202	9 (5)	9 (5)	*0.393*	6 (4)	7 (4)	11 (5)*	*< 0.001*
PCS- Magnification (0–12/12)	203	6 (4)	6 (4)	*0.175*	4 (3)	5 (3)	7 (3)*	*< 0.001*
PCS- Helplessness (0–24/24)	202	10 (7)	10 (7)	*0.239*	6 (5)	8 (7)	12 (6)*	*< 0.001*
OMPQ total score (0–210/210)	203	53 (18)	54 (19)	0.500	46 (11)	49 (20)	65 (12)*	*< 0.001*
OMPQ expectations of recovery (0–10/10)	210	4 (3)	4 (3)	0.899	3 (2)	4 (3)	6 (3)*	*< 0.001*
General Health								
SF −12: MCS (0–100/100)	209	43 (13)	43 (12)	0.887	49 (13)	43 (13)	38 (11)*	*< 0.001*
SF −12: PhCS (0–100/100)	209	33 (9)	33 (9)	0.790	37 (9)	34 (10)	28 (6)*	*< 0.001*
EQ5D 3 L								
VAS pre-accident (0–100/100)	211	85 (12)	82 (14)	0.016*	86 (10)	85 (10)	84 (14)	*0.786*
Pre-accident (0–1/1)	210	1 (0.1)	1 (0.2)	0.512	1 (0.1)	1 (0.1)	1 (0.2)	*0.213*
Post-accident (0–1/1)	211	0.3 (0.3)	0.3 (0.4)	0.640	0.5 (0.3)	0.3 (0.4)	0.1 (0.3)*	*< 0.001*
Psychological								
IES-R total score (0–88/88)	210	39 (24)	38 (22)	*0.860*	33 (22)	33 (23)	53 (21)*	*< 0.001*
IES-R total sub scale score (0–12/12)	213	5.2 (3.4)	4.5 (3.4)	*0.103*	5.1 (3.6)	5.2 (3.3)	5.1 (3.5)	*0.963*
IES-R - Avoidance (0–4/4)	214	1.7 (1.2)	1.6 (1.1)	*0.521*	1.7 (1.3)	1.7 (1.1)	1.6 (1.2)	*0.911*
IES-R - Intrusion (0–4/4)	214	1.7(1.2)	1.5 (1.2)	*0.147*	1.6 (1.2)	1.8 (1.2)	1.7 (1.3)	*0.854*
IES-R - Hyperarousal (0–4/4)	204	1.8 (1.2)	1.5 (1.1)	0.010*	1.9 (1.2)	1.8 (1,1)	1.8 (1.2)	*0.774*
PDS - Hyperarousal (0–15/15)	204	8 (4)	8 (4)	*0.909*	6 (5)	7 (4)	10 (3)*	*< 0.001*
DASS total score (0–63/63)	211	20 (18)	21 (19)	*0.651*	14 (16)	17 (16)	29 (18)*	*< 0.001*
DASS - Depression (0–21/21)	211	6 (6)	6 (6)	*0.632*	4 (6)	5 (5)	9 (7)*	*< 0.001*
DASS - Anxiety (0–21/21)	204	6 (6)	7 (7)	*0.212*	4 (5)	4 (5)	9 (6)*	*< 0.001*
DASS - Stress (0–21/21)	204	8 (7)	8 (7)	*0.896*	6 (7)	7 (6)	12 (6)*	*< 0.001*

*Abbreviations*: *LOS* Length of stay, *NDI* Neck Disability Index, *NRS* Numeric Rating Scale, *PCS* Pain Catastrophising Scale, *OMPQ* Orebro Musculoskeletal Pain Questionnaire, *SF-12* Short-form 12 general health questionnaire, *EQ5D 3 L* Euro Quality of Life – 5 Dimensions - 3 Levels, *IES-R* Impact of Events Scale – revised, *PTSD* Post-traumatic Stress Diagnostic Scale, *DASS* Depression, Anxiety and Stress scale

Post hoc significance: ****low vs. med vs. high; *high vs. low and med; **med vs. low and high. *p* > 0.05

**Table 3:** Comparison of patient-reported EBC, therapeutic relationship and resource distribution between risk groups

Variables	N	All participants (*n* = 160)	Low risk (*n* = 24)	Medium risk (*n* = 79)	High risk (*n* = 48)	*P*-values
Diagnosis received, n (%)	159	112 (70%)	12 (50%)	58 (74%)	33 (69%)	0.080
WAD		59 (37%)	7 (58%)	29 (50%)	17 (52%)	
Other		53 (31%)	5 (42%)	29 (50%)	16 (49%)	0.871
Canadian Cervical Spine rule, n (%)						
Total x-rays required and received	152	109 (68%)	13 (59%)^l^*	52 (69%) ^l^*	40 (87%)	0.026
Total x-rays required and not received	152	30 (19%)	8 (36%)^l^*	14 (19%) ^l^*	5 (11%)	0.042
Total x-rays not required and received	152	6 (4%)	1 (5%)	5 (7%)	0 (0%)	0.206
Total x-rays not required and not received	152	7 (4%)	0 (0%)	4 (5%)	1 (2%)	0.410
Total cases of Cervical Spine rule correctly applied	152	116 (73%)	13 (59%)	56 (75%)	41 (89%)*	0.018
Management of high – risk individuals						
Number referred, n (%)	159	45 (28%)	3 (13%)	22 (28%)	17 (36%)	0.124
Time to referral, weeks, mean (SD)	40	4 (4)	2 (2)	4 (3)	5 (5)	0.477
Referral appropriate, n (%)	144	30 (19%)	21 (88%)***	45 (60%)	16 (36%)	< 0.001
Therapeutic relationship score (9–45/45), mean (SD)	131	40 (6)	38 (7) ^l^*	42 (4) ^l^*	39 (7)	0.031
NDI sum score (0–50/50), mean (SD)	159	13 (11)	5 (7)****	10 (9)****	22 (10)****	< 0.001
GPE (−5 to + 5), mean (SD)	158	3 (2)	4 (2)	3 (2)	1 (2)*	< 0.001
Resources booklet received, n (%)	159	28 (17%)	2 (8%)	12 (15%)	8 (19%)	0.512
Resources booklet received from, n (%)	29					
Insurer		6 (20%)	0 (0%)	2 (15%)	2 (22%)	
Health practitioner		11 (40%)	2 (67%)	3 (23%)	6 (67%)	
SIRA		9 (31%)	1 (33%)	6 (46%)	1 (11%)	
Other		3 (10%)	0 (0%)	2 (15%)	0 (0%)	0.295
WAD-Evidence tool score (range − 85-165), mean (SD)	160	61 (28)	59 (17)	60 (28)	63 (32)	0.931

*Abbreviations*: *GPE* Global perceived effect, *NDI* Neck Disability Index, *SIRA* State Insurance Regulatory Authority, *WAD* Whiplash associated disorder

Post hoc significance: ****low vs. med vs. high; *high vs. low and med; ***low vs. med and high; ^l^*low vs. medium. *p* <  0.05. NB. Percentage scores for ‘all participants’ are calculated as a percentage of the 160 participants completing interviews

### Baseline characteristics of participants with whiplash

Fifty-five percent of participants with whiplash were female, with a mean age of 42 years (Table 2). The majority (87%) were recruited from hospital emergency departments. Four percent were recruited from physiotherapy practices, 4% from the PIR database, and 5% from the CAS database. Correlation by centre for variables of interest was examined using correlated data models and found to be negligible (working correlation < 0.05).

Seventeen percent of the cohort was classified as low-risk, 51% as medium-risk, and 32% as high-risk. Individuals in the high-risk group were higher in age (*p* <  0.001) and presented to hospital more frequently post-injury (*p* = 0.040). A significantly greater number were single (*p* <  0.001), and not currently in paid employment (*p* <  0.001). They also had a higher mean body mass index (BMI) (*p* = 0.038; Table 2). Those lost-to-follow-up at 3 months had a shorter initial hospital stay, lower EQ5D3L pre injury scores and lower IES-R hyperarousal sub scale scores (e.g. mean (SD) all: 1.8(1.2), lost to follow-up: 1.5(1.1); *p* = 0.010; Table 2). No other differences were observed in baseline characteristics between all participants and those lost-to-follow-up.

#### Participants with whiplash: outcomes at 3 months

Ninety-three percent of the cohort sought care from a primary care provider following their injury. General practitioners (GP) were most frequently consulted (84%), followed by physiotherapists (60%), chiropractors (13%), massage therapists (6%), psychologists (3%) and osteopaths (3%).

### Compliance with content messages

According to participant self-report, the Canadian C-Spine Rule was applied correctly more frequently for individuals at high-risk than for individuals at low- and medium-risk (59% low-risk, 75% medium-risk, 89% high-risk; *p* = 0.018; Table 3). Across the cohort, only 4% of participants received unnecessary imaging. However, 19% of participants interviewed reported at least one risk factor necessitating x-ray, though had not received an x-ray.

**Fig. 2:**
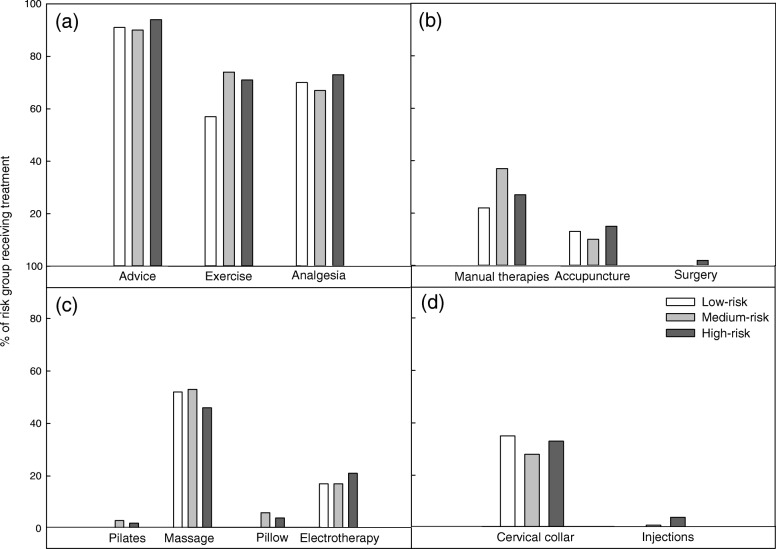
Participants with whiplash: Proportion of participants with whiplash reporting treatment, stratified by risk of non-recovery. **a** recommended treatments, **b** treatments not routinely recommended, **c** treatments with no evidence for or against their use, **d** treatments not recommended

The majority of the cohort (> 90% across all risk groups) received recommended active treatments. A large proportion of individuals received treatments with no evidence for or against their use (e.g. massage: 52% low-risk, 53% medium-risk and 46% high-risk; Fig. 2). There was no significant variation in the proportions of individuals between groups receiving treatments across all recommendation categories (Fig. 2).

### Compliance with process messages

**Fig. 3:**
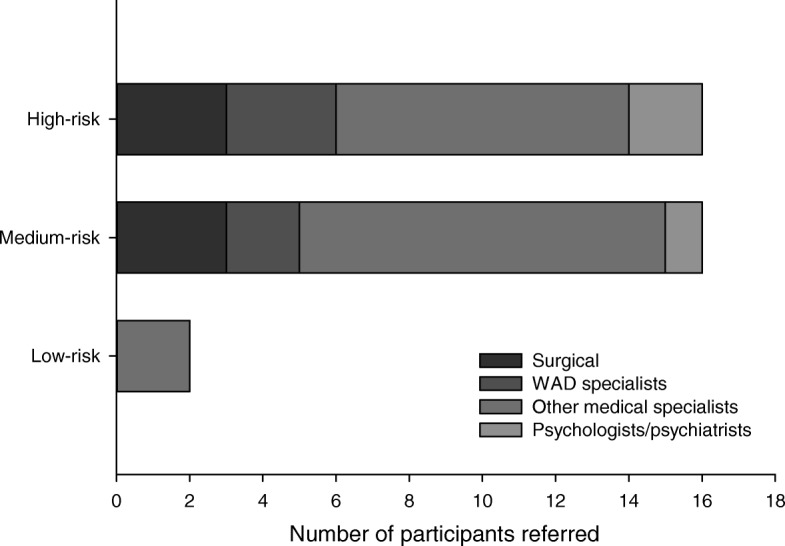
Proportion of participants with whiplash referred to specialists of different disciplines at 3 months post injury

The mean number of treatments was similar between groups (mean (SD) low-risk: 8(6), medium-risk: 9(9), high-risk: 10(7); *p* = 0.54). Only 49% of individuals at high-risk received the appropriate number of treatments (e.g. 6–16 treatments; Table 1). Similarly, the proportion of individuals that were referred for a specialist opinion across risk groups did not vary, with a very low proportion of individuals at high-risk appropriately referred (88% low-risk, 60% medium-risk, 36% high-risk appropriately referred; *p* <  0.001; Table 3). The majority of those referred were referred to traditional medical specialists including rheumatologists and neurologists (Fig. 3).

WAD-Evidence tool scores ranged from − 10 to 135 and did not vary between risk groups (mean (SD) low risk: 59(17), medium-risk: 60(28), high-risk: 63(32); *p* = 0.931; Table 3), suggesting similar levels of compliance with guideline recommendations.

### Recovery and therapeutic relationship

**Fig. 4:**
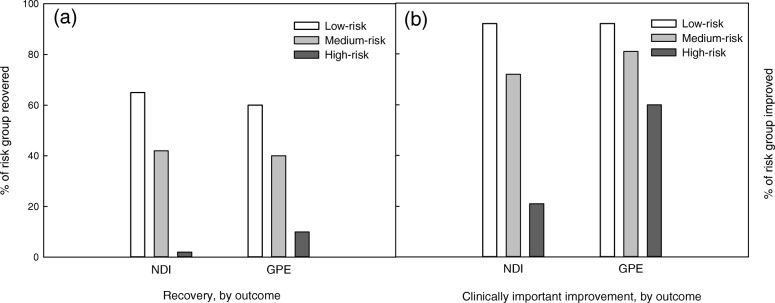
Recovery and clinically important improvements at 3 months in participants with whiplash. **a** proportion of individuals recovered at 3 months, and **b** proportion of individuals experiencing clinically relevant improvement at 3 months, stratified by risk of non-recovery

Self-reported disability due to neck pain significantly increased with risk status (mean NDI/50 (SD) low-risk: 5(7), medium-risk: 10(9), high-risk: 22(10), *p* <  0.001; Table 3). Similarly the mean GPE was significantly poorer for medium- and high-risk groups compared with low risk (mean (SD) GPE range − 5 to + 5: low-risk: 4(2), medium-risk: 3(2), high-risk: 1(2); *p* <  0.001; Table 3). Approximately 35% of the cohort was recovered at 3 months, with the proportion of individuals recovered reducing significantly with risk status. Fifty-eight percent of the cohort had minimal neck disability at three-months, and 76% experienced a minimal clinically important change in perceived recovery (Fig. 4).

The therapeutic relationship was high in all groups (mean (SD) low-risk: 38 (7), medium-risk: 42 (2), high-risk 39 (7); Table 3). Seventy-five percent of participants had scores of ≥38/45 (e.g. 85% total score), and this did not vary between risk groups (n (%) low risk: 12 (76%), medium-risk: 50 (82%), high-risk: 24 (67%); *p* = 0.169).

#### Primary HCPs: outcomes at 3 months

Details were received for 123 primary HCPs, of which 93 were contactable. Of those contactable, 53 completed the 3-month questionnaire. Reasons for difficulties in contacting and obtaining questionnaires from primary HCPs are shown in Fig. 1.

### Compliance with content messages

**Fig. 5:**
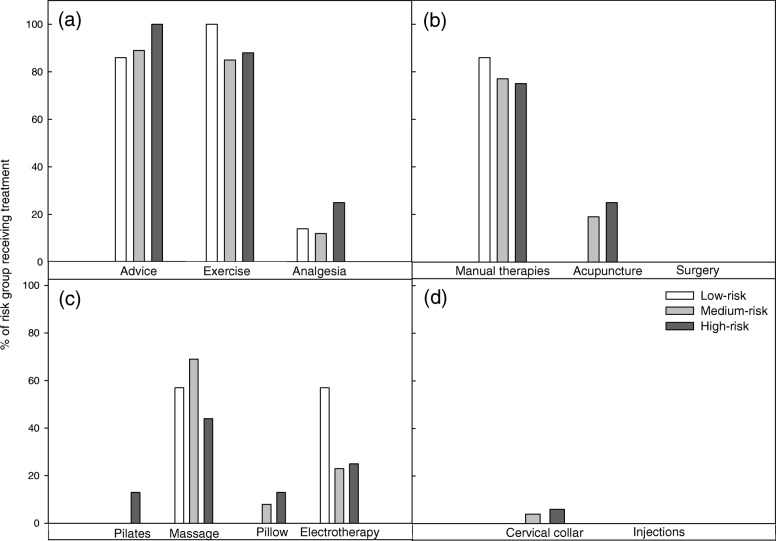
Primary Health Care Providers: Proportion of Primary HCP reporting provision of treatment, stratified by patient risk of non-recovery. **a** recommended treatments, **b** treatments not routinely recommended, **c** treatments with no evidence for or against their use, **d** treatments not recommended

Primary HCPs reported that the Canadian C-spine rule was correctly applied to a large proportion of their patients, across all risk groups (43–75%; Table 4). They also reported that a high proportion of their patients were provided with recommended treatments (e.g. advice 86–100%). Manual therapy and manipulation was also frequently prescribed (75–86%; Fig. 5) across risk groups. There was no difference in the provision of care between risk groups.

**Table 4:** Comparison of primary HCP-reported evidence–based treatment provided, therapeutic relationship and resource distribution between risk groups

Variables	N	All participants (*n* = 53)	Low risk (*n* = 7)	Medium risk (*n* = 26)	High risk (*n* = 16)	*P*-value
Diagnosed received, n (%)	53	43 (81%)	5 (71%)	22 (85%)	13 (81%)	*0.725*
WAD (grade unspecified)		14 (35%)	1 (20%)	6 (27%)	7 (54%)	
WAD I		2 (5%)	1 (20%)	1 (5%)	0 (0%)	
WAD II		12 (30%)	2 (40%)	8 (36%)	2 (15%)	
WAD III		2 (5%)	0 (0%)	2 (9%)	0 (0%)	
Other		10 (25%)	1 (20%)	5 (23%)	4 (31%)	*0.412*
Practitioner–assessed risk level, n (%)	53					
Low		29 (55%)	6 (86%)	14 (54%)	8 (50%)	
Medium		18 (34%)	0 (0%)	10 (39%)	6 (38%)	
High		6 (11%)	1 (14%)	2 (8%)	2 (13%)	*0.377*
Total cases of risk assessed correctly, n (%)	42	14 (26%)	6 (86%)***	10 (39%)	2 (13%)	*0.004*
Canadian Cervical Spine rule, n (%)						
Total X-rays required and received	52	35 (66%)	3 (43%)	20 (77%)	12 (75%)	*0.194*
Total X-rays required and not received	52	14 (26%)	4 (57%)	5 (19%)	3 (19%)	*0.095*
Total X-rays not required and received	52	2 (4%)	0 (0%)	0 (0%)	1 (6%)	*0.349*
Total X-rays not required and not received	52	1 (2%)	0 (0%)	1 (4%)	0 (0%)	*0.637*
Cases of Cervical Spine rule correctly applied, n (%)	52	36 (68%)	3 (43%)	21 (80%)	12 (75%)	*0.129*
Appropriate treatment						
Number of treatments, mean (SD)	53	12 (8)	6 (4)***	10 (8)	15 (9)	*0.035*
Number of treatments appropriate, n (%)	43	14 (26%)	2 (33%)	8 (36%)	11 (79%)	*0.634*
Management of high – risk individuals						
Number referred^, n (%)	53	19 (36%)	0 (0%)^l^*	8 (31%)	9 (56%)	*0.028*
Medical	53	5 (9%)	0 (0%)	3 (12%)	2 (13%)	*0.626*
Surgical	53	5 (9%)	0 (0%)	2 (8%)	2 (13%)	*0.597*
Pain specialist	53	6 (11%)	0 (0%)^l^*	1 (4%)	5 (31%)	*0.018*
Psychologist or Psychiatrist	53	5 (9%)	0 (0%)	1 (4%)	3 (19%)	*0.160*
Physiotherapy specialist	53	3 (6%)	0 (0%)	1 (4%)	1 (6%)	*0.781*
Other	53	1 (2%)	0 (0%)	1 (4%)	0 (0%)	*0.637*
Mean time to referral, weeks, mean (SD)	17	5 (4)	–	6 (5)	5 (4)	*0.746*
Referral appropriate, n (%)	47	38 (72%)	7 (100%)	22 (96%)	8 (50%)*	*< 0.001*
Therapeutic relationship score (9–45/45), mean (SD)	53	39 (4)	37 (5)	40 (4)^m^*	36 (8)	*0.046*
Awareness of Australian whiplash guidelines, n (%)	53	40 (76%)	5 (71%)	18 (69%)	13 (81%)	*0.687*
Name of guidelines, n (%)	14					
Described Australian whiplash guidelines		23 (43%)	3 (60%)	10 (52%)	8 (73%)	
Described other		10 (19%)	2 (40%)	6 (32%)	2 (18%)	
Did not describe any guidelines		6 (11%)	0 (0%)	2 (13%)	1 (9%)	*0.715*
Guidelines informed patient management, n (%)	53	33 (62%)	5 (71%)	15 (60%)	10 (63%)	*0.859*

*Abbreviations*: *GPE* Global perceived effect, *NDI* Neck Disability Index, *SIRA* State Insurance Regulatory Authority, *WAD* Whiplash associated disorder

Post hoc significance: *high vs. low and med; ***low vs. med and high; ^l^*low vs. med; ^m^*med vs. high. *p* < 0.05. NB. Percentage scores for ‘all participants’ are calculated as a percentage of the 53 primary health care providers completing interviews. ^Participants with whiplash may be referred to more than one specialist practitioner

### Compliance with process messages

Primary HCPs reported 100% of patients at low-, and 96% of patients at medium-risk to have received appropriate referral, compared to 50% of patients at high-risk (*p* <  0.001; Table 4). The ability of primary HCPs to correctly identify patients at high risk of non-recovery was poor (86% low-risk, 39% medium-risk, 13% high-risk correctly identified; *p* = 0,004; Table 4).

### Therapeutic relationship

Therapeutic relationship was also reported to be high across all risk groups according to primary HCPs (Mean > 36/45; Table 4).

### Inherent bias in practitioner reporting

It is important to consider the influence of selection and recall bias associated with the means by which practitioners were recruited to the study, and the way in which their data were collected. Practitioners had the option to participate and were asked to recall the treatments provided to their patients in retrospect. It is therefore possible that recruitment may have favoured practitioners with greater confidence in their treatment. Practitioners may have also recalled the provision of their treatment as more adherent with the guidelines than it may in fact have been.

#### Prediction of recovery

The univariate logistic regression analyses revealed an association between recovery and the known predictors of recovery in WAD (e.g. CPR, expectations of recovery) (Table 5). The EQ5D3L pre-injury score, and the EQ5D3L VAS score, were not significantly associated with recovery.

**Table 5:** Univariate logistic regression analysis showing relationship between previously known predictors of recovery, and recovery (GPE and NDI) at 3-months post injury

	GPE					NDI				
	OR	95% CI		Nagelkerke R^2^	*P*-value	OR	95% CI		Nagelkerke R^2^	*P*-value
CPR (low-risk vs high-risk)	13.067	3.746	45.579	.198	0.001*	82.5	9.516	715.277	.337	0.000*
Expectations of recovery	.768	.669	.881	.147	< 0.0001*	.668	.569	.785	.272	0.000*
Baseline pain intensity	.641	.520	.789	.190	< 0.0001*	.630	.510	.778	.201	0.000*
IES	.975	.961	.990	.106	0.001*	.967	.952	.983	.174	0.000*
EQ5D3L pre	15.843	.748	335.672	.034	*0.076*	10.780	.532	218.251	.026	*0.121*
EQ5D3L post	9.450	3.196	27.938	.168	< 0.0001*	22.026	6.451	75.202	.268	0.000*
EQ5D3L VAS	1.015	.983	1.048	.008	*0.376*	1.018	.985	1.052	.011	*0.283*
SF-12 physical	1.076	1.034	1.119	.132	< 0.0001*	1.103	1.055	1.152	.207	0.000*
SF-12 mental	1.078	1.043	1.114	.212	< 0.0001*	1.079	1.043	1.116	.213	0.000*
DASS total score	.957	.934	.980	.137	< 0.0001*	.943	.918	.969	.210	0.000*
DASS depression sub scale	.894	.834	.958	.107	0.002*	.844	.776	.917	.195	0.000*
PCS	.957	.930	.984	.097	0.002*	.934	.905	.964	.197	0.000*

*Abbreviations*: *CI* Confidence interval, *CPR* Clinical prediction rule, *DASS* Depression Anxiety Stress Scale, *EQ5D3L* Euro Quality of Life – 5 dimensions - 3 levels, *IES-R* Impact of Events Scale – revised, *OR* Odds ratio, *PCS* Pain catastrophising scale, *SF-12* Short Form Health Survey 12, *VAS* Visual analogue scale. * statistically significant at *p* < 0.05

The first multivariate logistic regression model aimed to determine predictors of GPE. This model contained the CPR, SF-12 mental component summary (MCS) and SF-12 physical component summary (PhCS). The CPR was a good predictor of recovery within this cohort, with individuals at medium-risk of non-recovery 35% less likely to recover than those at low-risk. Individuals at high-risk of non-recovery were 80% less likely to recover than those at low-risk. The model correctly classified 75% of participants, and when therapeutic relationship was added, the model correctly classified 78% of participants. Therapeutic relationship was associated with higher odds of recovery of between 2 and 34% (OR 1.17, 95% CI 1.018–1.340), representing a potentially clinically significant improvement. The WAD-Evidence tool score did not contribute significantly to the model (OR 0.999, 95% CI 0.983–1.012, *p* = 0.881).

The second model measuring recovery via NDI scores contained the CPR, EQ5D3L post injury, and OMPQ expectations of recovery. Again, the CPR was an important predictor of recovery, with individuals at medium-risk 53% less likely to recover than individuals at low-risk. Further, individuals at high-risk of non-recovery were 69% less likely to recover than individuals at low-risk. The model correctly classified 81% of participants. When therapeutic relationship was added to the model, it correctly classified 83% of participants. It was again associated with higher odds of recovery, between 11 and 62%, and potentially clinically significant (OR 1.345, 95% CI 1.118–1.617; Table 6). WAD-Evidence tool score did not contribute significantly to the model (OR 1.008, 95% CI 0.990–1.026). The interaction between WAD-Evidence tool score and therapeutic relationship was also assessed, and was non-significant for both GPE (*p* = 0.301) and NDI (*p* = 0.905) outcomes.

**Table 6:** Multivariate logistic regression predicting likelihood of recovery at 3 months post injury for GPE and NDI outcomes

Outcome	Predictor variables	OR	95% CI		*p*-value	Nagelkerke R^2^
GPE	CPR (medium-risk vs. low-risk) CPR (high-risk vs. low-risk)	.646 .197	.226 .050	1.849 .781	*0.415* 0.021*	
	SF-12 mental	1.070	1.032	1.109	0.000*	
	SF-12 physical	1.056	1.009	1.105	0.019*	.364
	*Therapeutic relationship*	*1.168*	*1.018*	*1.340*	0.026*	*.384*
	*WAD-Evidence tool score*	*.999*	*.983*	*1.014*	*0.881*	*.364*
NDIa	CPR (medium-risk vs. low-risk) CPR (high-risk vs. low-risk)	.462 .31	.155 .003	1.380 .290	*0.167* 0.002*	
	EQ5D3L post	8.267	2.038	33.530	0.003*	
	OMPQ expectations	.780	.645	.944	0.011*	.496
	*Therapeutic relationship*	*1.345*	*1.118*	*1.617*	0.002*	*.599*
	*WAD-Evidence tool score*	*1.008*	*.990*	*1.026*	*0.380*	*.500*

*Abbreviations*: *CI* Confidence interval, *CPR* Clinical prediction rule, *EQ5D3L* Euro Quality of Life – 5 dimensions - 3 levels, *OR* Odds ratio, *OMPQ expectations* OMPQ expectations of recovery, *SF-12* Short Form Health Survey 12; *statistically significant at *p* < 0.05

## Discussion

Evidence-based care, measured using the WAD-Evidence tool was received by, and provided to, the majority of individuals with acute whiplash in this inception cohort. Key recommendations relating to content of care and provision of recommended active treatments appeared well followed. However, guideline recommendations for a reduction in the provision of passive treatments, and recommendations for the process of care including risk identification and appropriate referral, were poorly followed. Our study also found that the CPR and the therapeutic relationship were accurate at identifying recovery at 3-months post injury. However, the provision of EBC measured using the WAD-Evidence tool was not associated with recovery. Recommendations regarding future implementation strategies will be discussed.

The baseline characteristics of this inception cohort were typical to those of previous WAD cohorts, with the exception of gender distribution. Our cohort comprised relatively fewer females (55%) compared to other cohorts, which are typically 60–62%, [31, 43–46] though may be as high as 69%, female [4]. On further investigation, gender was not found to influence recovery, for both GPE and NDI outcomes. The vast majority of our cohort were recruited from hospital emergency departments and SIRA databases, with a combined refusal rate of 32%. This study was limited by the inability to compare demographics between those individuals that participated and those that declined participation, as retention of data for non-participating individuals was not possible.

The Canadian C-Spine rule was applied correctly for the majority (74%) of this cohort, with a very low rate (4%) of unnecessary imaging identified. This is consistent with findings from a multi-centre cluster randomised controlled trial, [47] where the rule was correctly applied for 83% of the cohort, with only 2% receiving unnecessary imaging. The trial identified a sensitivity rate of 100%. However, our study revealed that a moderate proportion (19%) of participants with whiplash did not receive an x-ray when indicated. When the indication(s) for receiving x-ray for these individuals were compared, the majority of our cohort (60%) reported they were unable to turn their head at least 45 degrees bilaterally. Although difficult to determine with certainty, physicians in the hospital emergency department would likely have assessed this risk factor objectively. It is therefore likely that the retrospective reporting of this risk factor by participants in our cohort may have increased the number of individuals testing false positive on the rule.

Provision of the guideline-recommended active treatments by primary HCPs was excellent. This suggests that the implementation of messages around the provision of these treatments have been effective. Previous implementation strategies undertaken in Australia to ensure these messages were implemented included the provision of online [7] and face-to-face [8, 9] education amongst insurers, [48] GP’s [7] and allied health practitioners [8, 9]. An uptake of > 80% of the activating information was reported amongst practitioners. Our data was collected 3 years after these strategies, suggesting that these messages have been retained. Therefore, a shift in focus from guideline-recommended activating treatments to those guideline recommendations less well implemented, is recommended.

A high proportion of this cohort received passive treatments that are not routinely recommended or have no evidence supporting their use, including massage (received by 50%) and manual therapy and manipulation (received by 33%). This recommendation was made on the basis of several trials highlighting a lack of evidence supporting the use of passive treatments including acupuncture, [49] manual therapy and manipulation, [50] and soft collars [51–53]. Our findings suggest that passive treatments are frequently applied for WAD, which is of significant concern, and supported by a recent cross-sectional survey in which 80% of Australian GPs identified manipulation as a recommended treatment [54]. Together with a reduction in the costs associated with the provision of unnecessary care, a reduction in the selection of passive treatment modalities may have benefits for the patient related to reduced dependency and improved self-efficacy.

Alternate strategies to reduce the provision of passive treatments are warranted. In other populations, guideline adherence has improved with concurrent patient and clinician education. Targeted patient education strategies, including practice-based and mailed household educational material, has reduced unnecessary antibiotic prescription for bronchitis by 40%, when run concurrently with clinician education [55]. Additional strategies, including computer generated, [56] interactive [57] reminders for clinicians and audit and feedback on professional practice delivered by superiors, [58] have also improved practice, though effects were generally small.

Organisation-targeted strategies may be more effective. In a single-site before-and-after study, [59] the Canadian C-spine rule was formally adopted as emergency department policy in a community teaching hospital in Melbourne, Australia. Staff were educated on the Canadian C-Spine rule in groups and individually over a 2-month period, and provided with a reminder card, which attached to their identification badge. The result was a 25% relative reduction in X-ray ordering over a 3-month post intervention period. Similar organizational implementation strategies with the potential to reduce the provision of passive treatments may include changes to injury compensation schemes. For example, the compulsory third party (CTP) insurance compensation schemes in NSW may be revised, to exclusively support active, recommended treatments for those injured.

Organization changes may also have an important role in improving practices related to process of care. Since their inception in 1999, the Australian whiplash guidelines have recommended care be provided according to risk of non-recovery. Specifically, individuals at lower risk should receive less intervention, and individuals at medium to high risk, more. We found no difference in the number of treatments received by individuals in different risk groups. The implementation of the validated CPR may be the first step in improving the process of care in WAD. Given the CPR was an accurate predictor of recovery in this cohort, its use in facilitating prognosis-matched management strategies by identifying risk of non-recovery should improve recovery, and is proposed in our recent trial protocol [20]. Mandating the use of the CPR by providing insurance-generated funding for rehabilitation only if risk status is identified, and capping the number of treatments based on risk level, could aid in facilitating prognosis-matched management practices.

Individuals at high-risk of non-recovery should also receive referral to a specialist clinician with expertise in the management of WAD. Our data indicated that very few individuals are referred (36% of individuals at high-risk), and largely to traditional medical specialists such as rheumatologists and neurologists (49% of those referred). The lack of recommended referral practices observed in the present study could be explained by the poor ability of primary HCPs to identify individuals at high-risk of non-recovery, and poor awareness of those with expertise in whiplash [60]. Previous strategies that have improved behaviour around referral included dissemination of guidelines with structured referral sheets attached, and specialist-run clinician education programs [61–63]. It is possible that similar strategies may be effective in improving referral practices in WAD, and further investigation is warranted.

Finally, the therapeutic relationship was identified as an important predictor of recovery following WAD, yet higher scores on the WAD-evidence tool were not. The relationship between therapeutic relationship and improved outcomes identified in this cohort is consistent with previous studies in populations including low back pain, [22, 64] post-traumatic stress disorder, [65] dissociative disorders [66] and schizophrenia [67]. Of note is that this is the first study to demonstrate a relationship between therapeutic relationship and recovery following whiplash injury, and represents a significant advancement toward a better understanding of recovery after whiplash injury.

The majority of the literature suggests that patients possessing a shared sense of goals and trust with their therapist can utilise these emotions in their recovery [66, 68]. Since the therapeutic relationship appears to have an important role in recovery, more so than the content of the care itself, the investment of time and effort on the part of the practitioner to develop and maintain a strong therapeutic relationship is warranted.

Our WAD-Evidence tool was not associated with recovery, suggesting either that receiving evidence-based care is not associated with outcome, or that our tool was not sensitive.

The known predictors of outcome in WAD are pain, disability and psychological factors [6, 17], as captured within the CPR. Since the CPR, along with the therapeutic relationship and additional outcome-specific predictor variables had already predicted a large proportion of the variance in recovery within our cohort, there is less remaining variance to explain through the addition of the WAD-Evidence tool scores. It is also important to consider that the majority of this cohort received recommended treatment, in terms of *content* of care. The issues identified with the provision of care related predominantly to the *process* of care, which may be more difficult to capture within an assessment tool.

## Conclusion

Messages related to the provision of guideline-recommended active treatments for acute WAD appear to have been successfully implemented. Therefore focus must shift to reducing the provision of passive treatments, and improving processes around the identification and appropriate management of at-risk individuals. Future work should investigate whether changes to CTP insurance funding for rehabilitation, interactive clinician education sessions, and the use of structured referral sheets disseminated within the Australian whiplash guidelines, may improve compliance.

## Supplementary information

**Additional file 1.** Questionnaire tool used to assess provision of evidence-based care.

## Data Availability

As these results are from a larger ongoing study, which will complete follow-up in approximately 24 months, we will not share the data at this stage. However, the dataset will be made publicly available on completion of the larger study. The author for correspondence may be contacted regarding any specific queries on the data presented in this report.

## Abbreviations

BMI: Body mass index
CAS: Claims advisory service
CPR: Clinical prediction rule
CTP: Compulsory third party
DASS: Depression, anxiety and stress scale
EBC: Evidence-based care
EQ 5D3L: European quality of life – 5 dimensions – 3 levels
GP: General practitioner
GPE: Global perceived effect
HCP: Health care practitioner
IES-R: Revised impact of events scale – revised
MCS: Mental component summary
NDI: Neck disability index
NRS: Numeric rating scale
NSW: New South Wales
OMPQ: Orebro musculoskeletal pain questionnaire
PCS: Pain catastrophising scale
PDS: Post-traumatic diagnostic scale
PhCS: Physical component summary
PIR: Personal injury register
PPV: Positive predictive value
SF-12: Short form-12
SIRA: State Insurance Regulatory Authority
VAS: Visual analogue scale
WAD: Whiplash associated disorder
WATOCI: Working Alliance Theory of Change Inventory
