# Supplementary material for: Evidence-based care in high- and low-risk groups following whiplash injury: a multi-centre inception cohort study
Source: BMC Health Serv Res. 2019 Nov 6;19:806. doi: 10.1186/s12913-019-4623-y (PMC6836463; doi:10.1186/s12913-019-4623-y)
Supplement: Supplementary file 1 — Additional file 1. Questionnaire tool used to assess provision of evidence-based care. [file 12913_2019_4623_MOESM1_ESM.docx]

**Additional file 1: Whiplash evidence-based questionnaire tool**

| **DIAGNOSIS** |
| --- |
| *‘In this first part of the interview, we would like to ask you a couple of questions relating to diagnosis of your injury’.*   1. **Have you been given a diagnosis about your neck-related injury? (*If no, go to question 3*)**   **□** Yes **□** No   1. **What was this diagnosis?  ___________________________________________________________________________  ___________________________________________________________________________**      1. **Was an X-ray of your neck taken?  □** Yes **□** No  3a. Was the estimated speed of the MVA >100 km/hr? **□** Yes **□** No  3b. Was the vehicle involved in a rollover or ejection? **□** Yes **□** No  3c. Was the vehicle a motorised recreational vehicle? **□** Yes **□** No  3d. Did you have pins and needles in your arms or legs? **□** Yes **□** No  3e. Was the vehicle hit by a bus or large truck? **□** Yes **□** No  3f. Were you able to walk at all times after the accident? **□** Yes **□** No  3g. Was your pain immediate (not delayed by minutes/hours/days)? **□** Yes **□** No  3h. Could you turn your head at least 45 degrees to the left and right? **□** Yes **□** No |

| **TREATMENT** |
| --- |
| *‘The next few questions relate to the treatment you have received for your whiplash injury’*   1. **Have you consulted any of the following practitioners?**   4a. General practitioner? **□** Yes **□** No   4b. Chiropractor? **□** Yes **□** No  4c. Osteopath? **□** Yes **□** No  4d. Massage therapist? **□** Yes **□** No  4e. Psychologist? **□** Yes **□** No  4f. Other    Specify: ________________________________________________________   1. **Of these, whom do you see most, or who is the main provider of your care?**  ___________________________________________________________________ 2. **Approximately how many sessions have you seen this practitioner for in total?** ___________________________________________________________________   If trouble answering the above, ask: ‘*how many sessions do you attend with this practitioner per week?*’     1. **Can you describe the treatment you received from this practitioner?**  ___________________________________________________________________  ___________________________________________________________________   This should be in the patient’s own words, if they are using medical jargon, ask: ‘*what did that involve?*’     1. **Did you receive any of the following treatments?** ***(Tick those applicable, and confirm those reported above as appropriate)*** 8a. Advice **□**   8b. Manual therapy/manipulation **□**   8c. Electrotherapy □   8d. Acupuncture □  8e. Pain-relieving medication □  8f. Injections □  8g. Surgery involving the neck □  8h. Exercises □   8i. Neck Pillow □  8j. Collar □   8k. Pilates/Feldenkrais or Alexander Technique □   8l. Massage □   8m. Other or additional treatment (specify below) □  __________________________________________________________________  __________________________________________________________________  __________________________________________________________________ 2. **Recommended exercises are provided in a booklet or DVD produced by the Motor Accidents Authority (MAA) of NSW. Did you receive this booklet or DVD? (*If no, go to question 11)* □** Yes **□** No 3. **How did you receive the booklet/DVD?**  10a. Sent by insurer **□**   10b. Sent by health practitioner **□**   10c. Sourced myself on the MAA’s website □   10d. Sent by the MAA □  10e. Other (specify) ___________________________________________________ |

| **REFERRAL** |
| --- |
| *‘The next few questions relate to referral to other practitioners’*   1. **Were you referred to a specialist of any kind? □** Yes **□** No ***(If no, go to question 12)***  11a. Medical (e.g. neurologist/rheumatologist) **□**   11b. Surgical **□**   11c. Pain specialist □   11d. Psychologist/psychiatrist □  11e. Physiotherapy specialist **□**   11f. Other (specify) ___________________________________________________ 2. **How long after your injury were you referred (weeks)?** ___________________________________________________________________ |

| **GLOBAL PERCEIVED EFFECT** |
| --- |
| *‘This last question is relates to how you feel you have progressed since your initial injury’.* **22. With respect to your whiplash injury, how would you describe yourself now compared to immediately after your accident? Please state a number on a scale of -5 to 5, where -5 means vastly worse, 0 means unchanged, 5 means completely recovered.**   \| ***-5*** \| ***-4*** \| ***-3*** \| ***-2*** \| ***-1*** \| ***0*** \| ***1*** \| ***2*** \| ***3*** \| ***4*** \| ***5*** \| \| --- \| --- \| --- \| --- \| --- \| --- \| --- \| --- \| --- \| --- \| --- \|   ***Vastly Unchanged Completely  worse recovered*** |

**Thank you for completing this survey.**
